# Hydroxyl-triggered fluorescence for location of inorganic materials in polymer-matrix composites†
†Electronic supplementary information (ESI) available: Detailed experimental materials, apparatus, experimental procedures and characterization data. See DOI: 10.1039/c7sc03897f


**DOI:** 10.1039/c7sc03897f

**Published:** 2017-10-16

**Authors:** Rui Tian, Jinpan Zhong, Chao Lu, Xue Duan

**Affiliations:** a State Key Laboratory of Chemical Resource Engineering , Beijing University of Chemical Technology , Beijing 100029 , China . Email: luchao@mail.buct.edu.cn ; Fax: +86 10 64411957 ; Tel: +86 10 64411957

## Abstract

There is a long-standing challenge to realize *in situ* visualization of incorporated inorganic materials in organic–inorganic composites in a post-labeling manner, owing to the lack of specific fluorescent organic dye molecules for targeting inorganic materials. Herein, we observe that the specific covalent B–O binding between the hydroxyl groups of inorganic materials and commercially available aggregation-induced emission (AIE)-active boronic acid could lead to the formation of highly emissive solid-state fluorescent composite materials. The hydroxyl-triggered luminescent probe may serve as a practical method for *in situ* location of incorporated inorganic materials in polymer-matrix composites by simply dipping the composite film in boronic acid-modified AIE solution. This present work offers a non-invasive avenue to locate inorganic materials which possess hydroxyl-groups in polymer-matrix composites, thereby developing a convenient screening strategy for assessing the advanced properties of composites. This strategy can also be extended to the targeted tracing of other inorganic materials with inherent and functionalized carboxyl, amino, sulfhydryl and other groups *via* tuning the binding affinity between the inorganic materials and luminescent molecules.

## Abstract 2

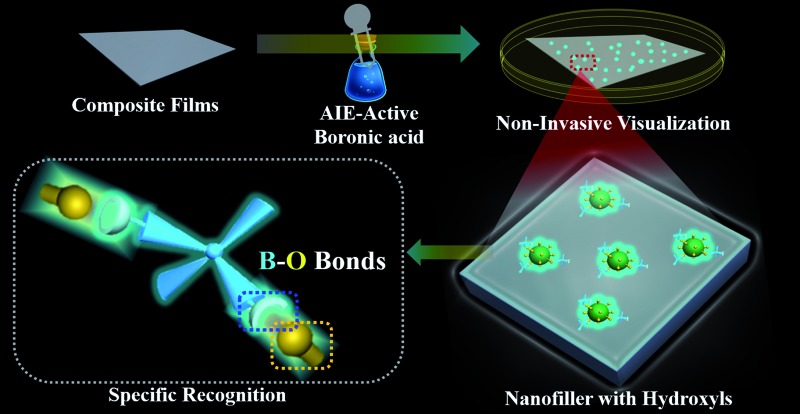
We present a locating technique for inorganic materials in polymer-matrix composites through a post-labeling approach based on specific covalent binding.

## Introduction

Incorporation of inorganic materials (*e.g.*, ions, molecules and supramolecules) into organic matrices has been regarded as an effective approach to advance functional composite materials.^1^–^3^ Therefore, the recognition and location of the incorporated materials within the matrices are essential not only for subsequent research but also for understanding the critical processing conditions necessary for quality control.^4^–^7^ Several techniques have been developed for the spatial location of incorporated materials, such as electron paramagnetic resonance, crystallographic and spectroscopic methods.^8^–^12^ Recently, fluorescence imaging techniques have become attractive alternatives for spatial visualization of individual incorporated materials in organic matrices. However, the established imaging techniques are mostly based on pre-modification of the inorganic materials with luminescent molecules before they are incorporated into the organic matrices.^13^,^14^ Actually, from an industry point of view, it is desirable to evaluate the inherent structural and functional behaviors of the incorporated materials in organic matrices without any addition of extraneous substances. Therefore, it is a topic of interest and significance to develop a simple location technique for inorganic materials in composites through a post-labeling process, instead of pre-modification.

Molecular targeted tracing techniques have been widely applied in biological fields, including tumor diagnostics, biomolecular detection and drug delivery.^15^,^16^ Such methods are mainly based on *in situ* visualization of the cell/biomolecules in the organism via labeling with luminescent molecules, keeping the inherent properties of the targeted molecules.^17^–^19^ These superiorities inspired us to introduce this type of technique into materials science, avoiding the changes to the inherent properties of the incorporated materials that occur in techniques with a pre-labeling aspect.

It has been reported that boronic acid-based covalent receptors possess a high targeting specificity towards hydroxyl groups on guests.^20^,^21^ Much effort has been made to sense carbohydrates by boronic acid in combination with fluorescence, colorimetry, and surface plasmon resonance.^22^–^24^ There is apparently no good reason to disregard the applications of hydroxyl-triggered luminescence techniques to recognize hydroxyl groups in materials science. This situation may be ascribed to the aggregation-caused quenching in solid-state material. Encouragingly, Sun and his co-workers recently employed an AIE-active tetraphenylethene-cored diboronic acid (TPEDB) for sensing glucose when the fluorogen was oligomerized with glucose through B–O bonds.^25^ Therefore, it is reasonable to anticipate the application possibilities of hydroxyl-triggered luminescence techniques in materials science using boronic acid-based covalent receptors.

**Scheme 1:**
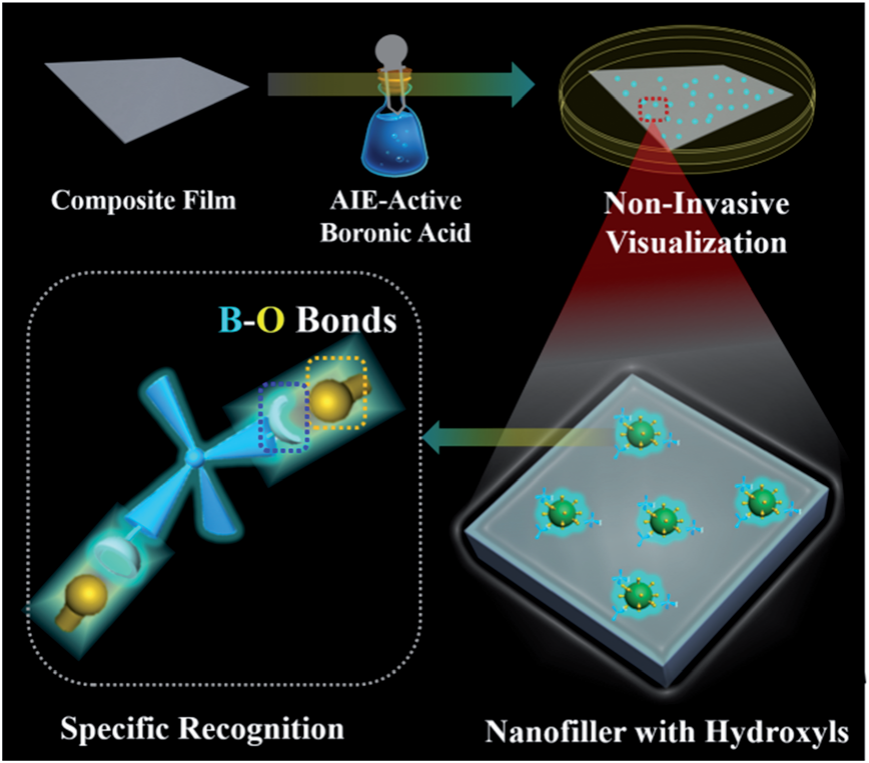
Schematic representation of hydroxyl-triggered fluorescence targeting techniques for layered materials in polymer-matrix composites.

Two-dimensional layered materials (*e.g.*, montmorillonite (MMT) and layered double hydroxides (LDHs)) are the most widely chosen additives to improve the properties of organic–inorganic composites.^26^,^27^ The abundant hydroxyl groups on the surfaces of two-dimensional layered materials have been used to construct a hydrogen-bond network between the hydroxide layers and the organic guests,^28^,^29^ facilitating the fabrication of various functional organic–inorganic composites.^30^,^31^ In this contribution, we observed that strong emissions are generated *via* specific B–O bonds between the hydroxyl groups of layered materials and AIE-active boronic acid. Furthermore, the visualization of incorporated MMT/LDHs in polymer-matrix composites has been implemented by simply dipping the composite film in AIE-active boronic acid solution without the aid of any pre-modification before the film formation (Scheme 1). The generality and simplicity of a post-labeling process are the highlights of our proposed approach: (1) the used AIE-active boronic acid reagent is commercially available; and (2) technicians could potentially adopt this approach without substantial training in chemistry. Therefore, the proposed strategy has great potential in the *in situ* screening of advanced properties for organic–inorganic composites.

## Results and discussion

**Fig. 1:**
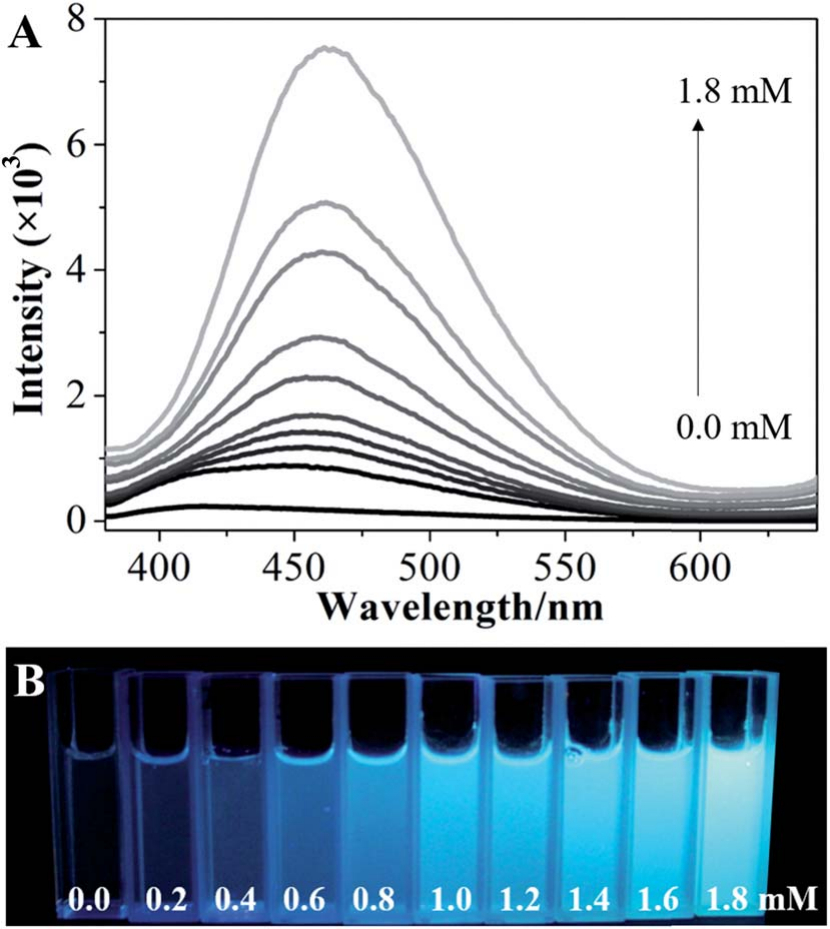
(A) Fluorescence emission spectra and (B) photos under UV light of TPEDB in the presence of the different concentrations of LDHs (in the range of 0.0–1.8 mM), respectively, *λ*_ex_ = 330 nm.

To explore the possibility of hydroxyl-triggered luminescence between AIE-active boronic acid and layered materials with hydroxyl groups, MgAl-LDHs were synthesized *via* a hydrothermal method (Fig. S1†). As a typical AIE compound with two boronic acid groups attached to the TPE core, TPEDB can be ionized and is molecularly soluble in alkaline medium.^32^ Fig. S2† shows that TPEDB can emit weak blue light at 415 nm with the excitation at 330 nm. However, the emissions of TPEDB were significantly intensified with the addition of LDHs (Fig. 1A). The strongest cyan emission for the TPEDB–LDH composites, which was ∼25-fold higher than that of pristine TPEDB, was achieved when the concentration of LDHs reached 1.8 mM. The greatly promoted fluorescence could also be visualized by photographs under UV irradiation (Fig. 1B). Moreover, the quantum yields of TPEDB-based samples have also been investigated, with the lowest value of 2.05% obtained for pristine TPEDB and the highest yield of 17.46% for TPEDB–LDHs (1.8 mM). These results indicated that the fluorescence intensity of TPEDB can be significantly enhanced in the presence of LDHs.

**Fig. 2:**
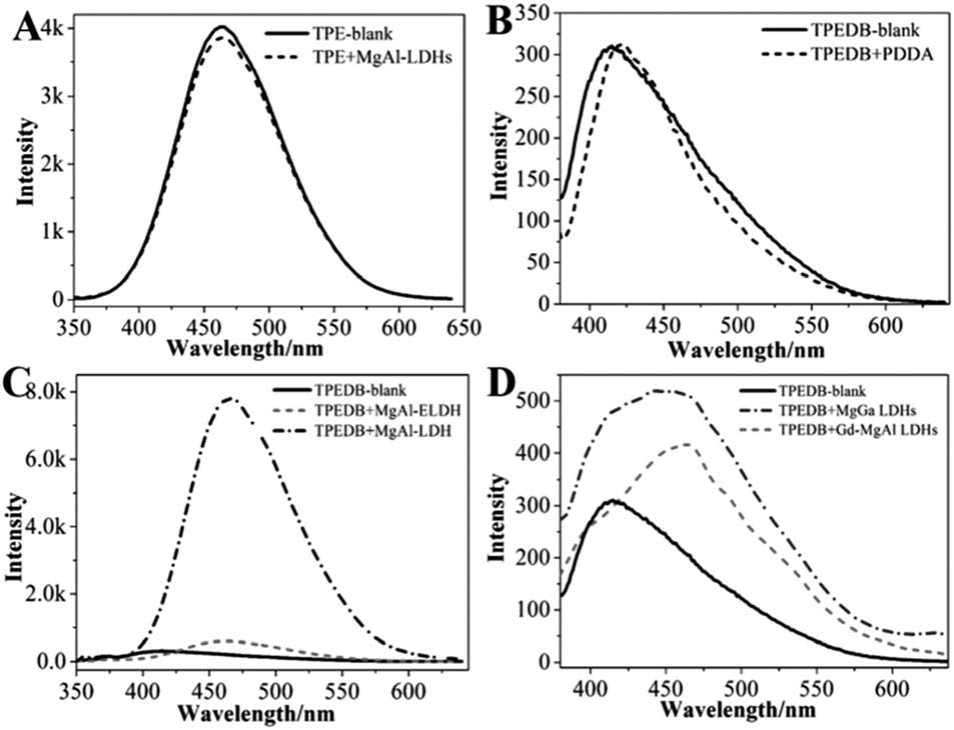
Fluorescence emission performances for (A) TPE molecules in the presence/absence of LDHs, (B) TPEDB in the presence/absence of PDDA, (C) TPEDB in the presence of LDHs and exfoliated LDH nanosheets, and (D) TPEDB in the presence of Gd-doped MgAl-LDHs and MgGa-LDHs, *λ*_ex_ = 330 nm.

A series of control experiments were performed to explore the specific B–O bonds between the hydroxyl groups of LDHs and the boronic acid of TPEDB. The precursors for the preparation of LDHs were studied, and the results showed that these precursors had no effect on the fluorescence emission intensities of TPEDB (Fig. S3†). On the other hand, the TPE core in the absence of boronic acid groups was also investigated; however, the emission spectra of the TPE remained the same after the addition of the LDHs (Fig. 2A). These results demonstrated that the LDH-enhanced TPEDB luminescence was not due to the adsorption of phenyl groups onto the surface of the LDHs. Moreover, the electropositive polymer poly(diallyldimethylammonium chloride) (PDDA) was added into the TPEDB solution. Interestingly, no obvious change in the fluorescence spectra was observed (Fig. 2B), indicating that the electrostatic interaction was not responsible for the TPEDB–LDH interactions. In conclusion, the above results implied that the binding affinity between the hydroxyl groups of LDHs and the boronic acid of TPEDB might exist.

In order to verify the binding affinity property, FT-IR measurements were conducted. Fig. S4A† shows the IR transmittance spectrum of LDHs, where the broad band at 3100–3600 cm^–1^ represents the hydroxyl groups and the peaks around 1350–1380 cm^–1^ stand for nitrate/carbonate in the interlayer of the LDHs.^33^,^34^ Unfortunately, the region of interlaminar nitrate/carbonate is coincident with the stretching of B–O bonds, which is in the range of 1310–1430 cm^–1^.^35^,^36^ As an alternative, another layered material with abundant hydroxyl groups, MMT, was also studied. As expected, obvious bands in the range of 1310–1430 cm^–1^ appeared for MMT after the addition of TPEDB (Fig. S4B†), indicative of B–O bond formation.^38^,^39^ We can draw the conclusion that the specific B–O bonds were formed between the boronic acid of TPEDB and hydroxyl groups of layered materials, which contributes to the restricted rotation of the phenyl units. Therefore, the non-radiation process of TPEDB was inhibited, resulting in the improved luminescence activity of TPEDB as illustrated in Fig. 1.^37^–^39^

It has been reported that carbohydrates with different structures (*e.g.*, number, relative distance and arrangement of hydroxyl groups) exhibit different binding affinities towards boronic acid.^21^–^23^ In this study, different LDHs and exfoliated LDH nanosheets (ELDH) with tunable hydroxyl groups were prepared. As seen in Fig. 2C, the ELDH could slightly increase the fluorescence intensity of TPEDB in comparison with the LDHs illustrated in Fig. 1. This phenomenon may be due to the hydroxyl sites occupied by formamide in order to maintain the monolayer state of the exfoliated LDH nanosheets.^40^ Furthermore, different metal ionic radii can change the lattice parameters of LDHs. Accordingly, the distance between adjacent hydroxyl groups is different.^41^–^44^ Herein, Gd-doped MgAl-LDHs and MgGa-LDHs were investigated (Fig. S5†).^44^ The results indicated that MgAl-LDH, with the closer hydroxyl arrangement, exhibited the larger fluorescence enhancement for TPEDB (Fig. 2C); however, the Gd-doped MgAl-LDHs and MgGa-LDHs, with remote hydroxyl distances, only displayed slight fluorescence enhancements (Fig. 2D). Therefore, the fluorescence intensities of TPEDB could be used for distinguishing the hydroxyl arrangement of different LDHs.

**Fig. 3:**
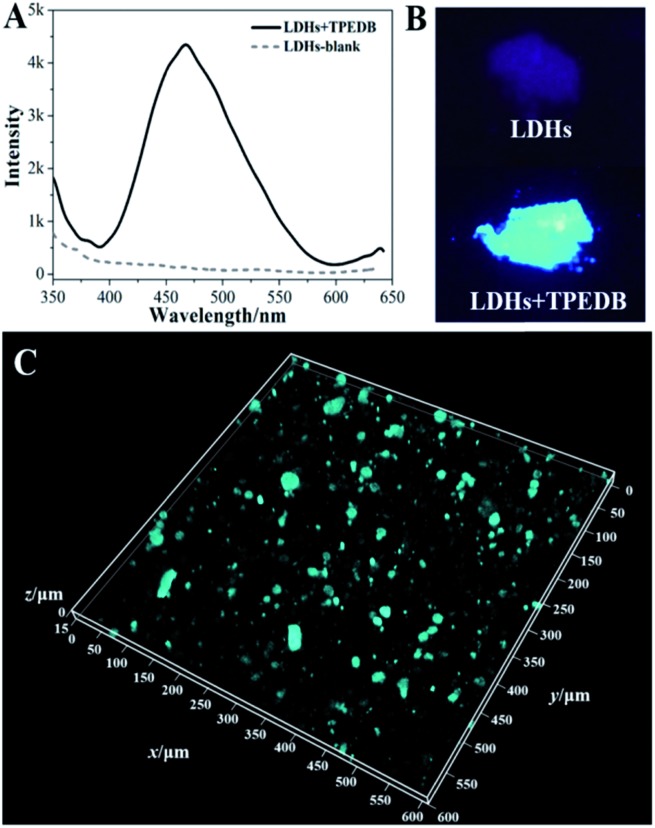
(A) Fluorescence spectra and (B) photos under UV light of LDHs before and after the addition of TPEDB; (C) a 3D-representation of fluorescence confocal microscopy images (600 × 600 μm^2^) of PE–5% LDH composite after staining with 100 μM TPEDB, the cyan colored patterns represent the stained LDH particles.

The precise and selective recognition of hydroxyl groups on the surfaces of LDHs by virtue of TPEDB emissions inspired us to develop a general method for targeted tracing of hydroxyl-containing layered materials in organic–inorganic composites by the confocal fluorescence microscopy (CFM) technique. To achieve this goal, we first investigated the binding affinity of TPEDB towards the LDH powder. The results showed that the non-emissive LDHs exhibited a strong cyan luminescence at 470 nm (Fig. 3A and B) with the addition of a certain amount of TPEDB (50 μL, 100 μM), indicative of the efficient binding between TPEDB and LDHs.

Next, LDHs were blended in polyethylene (PE) at a ratio of 5% and a stochastic area of the PE–5% LDH film was selected and then dipped into a 100 μM TPEDB solution for 10 min. As shown in Fig. S6,† great numbers of fluorescent cyan dots were captured to map the location of the LDHs in the PE matrix. However, no fluorescent dots appeared in the pure PE film in the absence of LDHs (Fig. S7†). Moreover, for spatial distribution characterization, three-dimensional (3D) imaging involving image collecting techniques at different depths along the *Z*-axis were adopted. Fig. 3C shows that the LDHs incorporated in the PE matrix can be clearly observed through fluorescence labeling by TPEDB in 3D (600 × 600 μm^2^). In comparison with the non-selective physical absorption process, these CFM results reflect the stable and specific location of LDHs in polymer-matrix composites by the proposed hydroxyl-triggered fluorescence method.

**Fig. 4:**
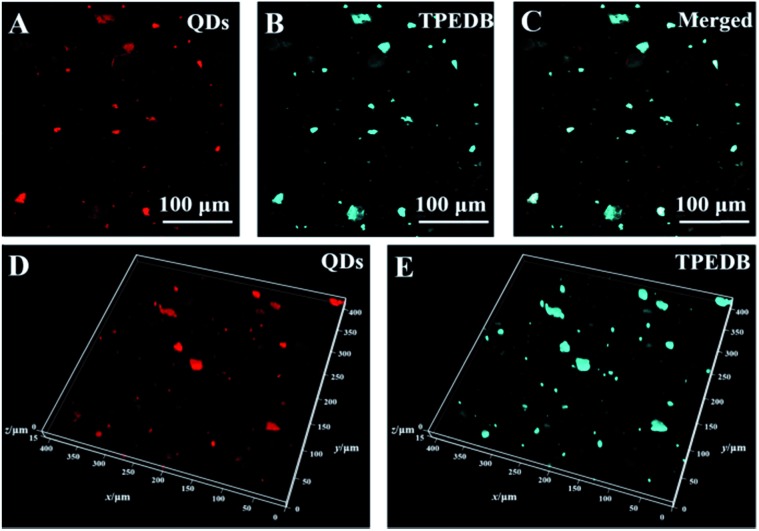
Fluorescence confocal microscopy images for co-stained PE–5% (QD@LDH) films for (A) red emission of pre-stained QDs, (B) cyan emission of post-stained TPEDB and (C) the image merged from panels A and B; 3D representations of co-staining techniques for (D) red emission of pre-stained QDs and (E) cyan emission of post-stained TPEDB. All fluorescence microscopy images were taken under a 405 nm laser.

To verify the accuracy of targeting hydroxyl groups on the surface of LDHs in polymer-matrix composites, two-color co-staining experiments were performed. In this work, the LDHs were pre-stained with red emission quantum dots (QDs) through electrostatic interaction to prepare the molded PE–5% (QD@LDH) film. The obtained PE–5% (QD@LDH) film was then dipped into the TPEDB solution to mark the LDHs *via* the B–O bonds. As depicted in Fig. 4A, the positions of the LDHs are represented as red dots of QDs in the PE matrix through a double-channel detection of CFM. On the other hand, the PE–5% (QD@LDH) film after post-staining by TPEDB showed cyan colored dots, as displayed in Fig. 4B. Interestingly, the merged picture of channel A and channel B reflected an almost perfect match between the QD pre-stained dots and TPEDB post-bonded particles (Fig. 4C). A 3D representation of this co-staining approach was also produced. It was observed that the coincident mapping of LDHs was acquired in composite films, even in different focal planes along the *Z*-axis (Fig. 4D and E). These results demonstrate that the proposed visualization strategy is a newly non-invasive platform to precisely target and trace the incorporated LDHs in polymer-matrix composites.

We further investigated the universality of this proposed method for mapping the spatial distribution of inorganic layered materials with hydroxyl groups in polymer matrices. The other typical layered material, MMT,^45^–^47^ was investigated to verify the specific binding between hydroxyl groups and boronic acid. The strong emission of MMT appeared in the presence of TPEDB as a result of the formation of B–O bonds (Fig. S8†). In addition, the incorporated MMT (5%) in PE can be distinctly screened through the precise match between the hydroxyl groups and boronic acid (Fig. S9†). To further assess this strategy, another polymer, polypropylene (PP), was also examined as a matrix for the LDH/MMT. Excitingly, the binding selectivity and molecular recognition were reproduced for the PP–5% LDH and PP–5% MMT films (Fig. S10–S12†), and the spatial distributions of the LDHs and MMT in PP were similar to those observed for PE. Therefore, our experimental data demonstrated the possibility of the *in situ* recognition and visualization of inorganic layered materials in polymer matrices through specific B–O bonds by a rapid and simple approach.

## Conclusions

In conclusion, we have demonstrated the presence of a strong solid-state emission of layered materials due to the formation of specific B–O bonds between hydroxyl groups on the surfaces of layered materials and AIE-active emissive boronic acid. This hydroxyl fluorescence location technique enabled us to establish a powerful imaging platform to precisely target and trace the incorporated inorganic materials in polymer-matrix composites. This post-labelling approach exhibited superiority in evaluating the inherent structural and functional behaviors of the inorganic–organic composites in comparison with the traditional pre-modification procedures. More importantly, such a unique fluorescent probe can be easily achieved by simply dipping polymer-matrix composite films in AIE-active boronic acid solution combined with high-resolution fluorescent imaging. Our strategy opens up new possibilities for optimization of critical processing conditions, while retaining the initial structural nature of polymer-matrix composites. This facile method can also be applied for the *in situ* targeted tracing of other inorganic materials by changing the binding affinity between the inorganic materials and AIE molecules.

## Conflicts of interest

There are no conflicts to declare.

## Supplementary Material

Supplementary information
