# Supplementary material for: Hydroxyl-triggered fluorescence for location of inorganic materials in polymer-matrix composites
Source: Chem Sci. 2017 Oct 16;9(1):218–22. doi: 10.1039/c7sc03897f (PMC5869289; doi:10.1039/c7sc03897f)
Supplement: Supplementary file 1 [file SC-009-C7SC03897F-s001.pdf]

Electronic Supplementary Material (ESI) for Chemical Science.
This journal is © The Royal Society of Chemistry 2017

Supporting Information for:

Hydroxyl-Triggered Fluorescence for Location of Inorganic Materials in Polymer–Matrix Composites

Rui Tian, Jinpan Zhong, Chao Lu,* and Xue Duan

State Key Laboratory of Chemical Resource Engineering, Beijing University of Chemical Technology, Beijing 100029, China

Experimental Section

Materials. Analytical-grade chemicals including $Mg(NO_3)_2 \cdot 6H_2O$, $Al(NO_3)_3 \cdot 9H_2O$, $Zn(NO_3)_3 \cdot 6H_2O$, $Ga(NO_3)_3 \cdot xH_2O$, $Gd(NO_3)_3 \cdot 6H_2O$, NaOH and poly(diallyldimethylammonium chloride) (PDDA) were purchased from Aladdin Chemical. Co. Ltd. Sodium montmorillonite (Na^+-MMT) with cation exchange capacity (CEC) values of 145 meq/100 g (from Nanocor, PGW grades) was used without further purification. Polyethylene (PE) powder with low density was purchased from Alfa Aesar. Polypropylene (PP) with the melt index of 12 g/10 min was purchased from Aladdin Chemical Co., Ltd. Tetraphenylethene-diboronic acid (TPEDB) was purchased from AIEgen Biotech Co., Limited and used without further treatment.

Preparation of $M^{II}M^{III}$-LDHs and TPEDB-LDHs composites. $M^{II}M^{III}$-LDHs were prepared according to a hydrothermal crystallization process.[1-3] For MgAl-LDH, $Mg(NO_3)_2 \cdot 6H_2O$ (0.075 mmol) and $Al(NO_3)_3 \cdot 9H_2O$ (0.025 mmol) were dissolved in 150 mL deionized water, and NaOH (0.5 M) was added dropwise until the pH of the suspension reached 8.5. The resultant suspension was further hydrothermally treated at 110 ^oC for 24h. Similarly, MgGa- and Gd-doped (30%) MgAl-LDHs were prepared with the final pH of 11. The as-prepared LDH suspensions were quantified according to the inductively coupled plasma (ICP) emission spectroscopy and prepared to different concentration for experiment.

The stock solution of TPEDB was firstly prepared by dissolving TPEDB into DMSO at a concentration of 10 mM.[4] Afterwards, 1 mL stock solution was added into the 99 mL carbonate buffer containing 1.120 g of K_2CO_3, 0.190 g $KHCO_3$ and 3 mL DMSO to prepare the TPEDB at 100 μM (pH=10.5). LDH suspensions (2 mL) with different concentration (0.0−3.6 mM) were added into TPEDB solution (2 mL, 100 μM) dropwise under continuous shaking, and the resulting suspensions

were shaken for another 10 min at room temperature. The pH of all the suspensions were maintained ~10 in the whole procedure to prepare TPEDB-LDH composites. Similarly, the control experiments were carried out in the same way, and the details are listed as followed:

Control samples	Amount of AIE molecules	Amount of references
TPE-MgAl-LDH	100 μM, 2 mL	3.6 mM, 2 mL
TPEDB-PDDA	100 μM, 2 mL	2.0 g/L, 2 mL
TPEDB-MgAl-ELDH	100 μM, 2 mL	3.6 mM, 2 mL
TPEDB-MgGa LDH	100 μM, 2 mL	3.6 mM, 2 mL
TPEDB-Gd-doped MgAl LDH	100 μM, 2 mL	3.6 mM, 2 mL

Preparation of organic-inorganic composite films. To fabricate PE-5% LDHs composite film, 1.0 g PE powder and 0.05 g MgAl-LDH powder were physically mixed and then melted at 120 ^{o}C. The resulting composite was molded at 120 ^{o}C for 1.5 min and then cooled to 30 ^{o}C. The PE-5% MMT composite was prepared according to the same procedure. Moreover, PP-5% LDHs and PP-5% MMT composites were prepared by blending 10.0 g PP and 0.5 g inorganic fillers in a heated double-roller mixer at 170 ^{o}C for 10 min, followed by molding at 170 ^{o}C for 2 min and the subsequent cooling procedure. For co-staining experiment, thioglycolic acid-capped CdTe quantum dots (QDs, red emission) were prepared according to the previous reference.[5] The LDHs (50 mg dispersed in 20 mL H_2O) were stirred with the addition of 0.02 mmol negatively-charged QDs, followed by washing, centrifugation and drying process. The QDs-modified LDHs were then mixed with PE and molded at 120 ^{o}C to obtain the PE-5% (QD@LDH) film.

Staining and hydroxyl location process. In order to stain the non-emissive organic-inorganic

composite films, stochastic areas in the films were selected and then dipped into the TPEDB (100 μM) for 10 min. The selected films were subsequently washed and ultrasonic treated with deionized water for another 10 min to remove the excess TPEDB molecules. Afterwards, the films were dried in a flow of nitrogen gas and prepared for sample characterizations.

Sample characterizations. X-ray diffraction (XRD) patterns of the LDHs were recorded using a Bruck (Germany) D8 ADVANCE X–ray diffractometer under the conditions: 40 kV, 50 mA, Cu K radiation (λ= 0.1541 nm) in the range from 5° to 65°. The morphologies of the LDHs were investigated using a scanning electron microscope (SEM Hitachi S-3500) with the accelerating voltage set at 20 kV. FT-IR spectra of flexible films were recorded on a Nicolet 6700 (Thermo Electron) in the range of 600-4000 cm^{-1}. The fluorescence spectra were performed on an F–7000 fluorescence spectrophotometer (Hitachi, Japan), the emission spectra were recorded in the range of 350−650 nm with the excitation wavelength of 330 nm. The quantum yields were recorded on an Edinburgh FLS 980 steady state spectrometer equipped with an integrating sphere. The elemental content of LDHs was determined by inductively coupled plasma (ICP) emission spectroscopy on a Shimadzu ICPS-7500 instrument. The fluorescence microscope images for stained organic-inorganic composite films were captured on a confocal laser scanning microscope (Leica TCS SP8) with the excitation wavelength of 405 nm, and the *XY* and *Z* resolutions to investigate the structures of the composite films are empirically estimated to be 200 nm and 600 nm, respectively.[6]

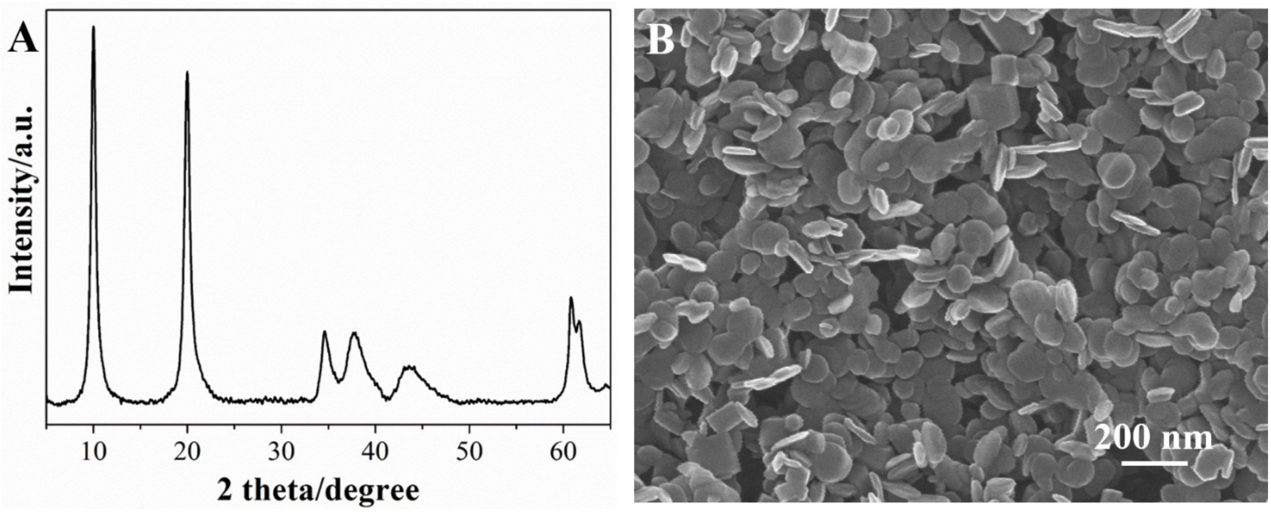

Fig. S1 (A) XRD pattern and (B) SEM image of MgAl-LDHs.

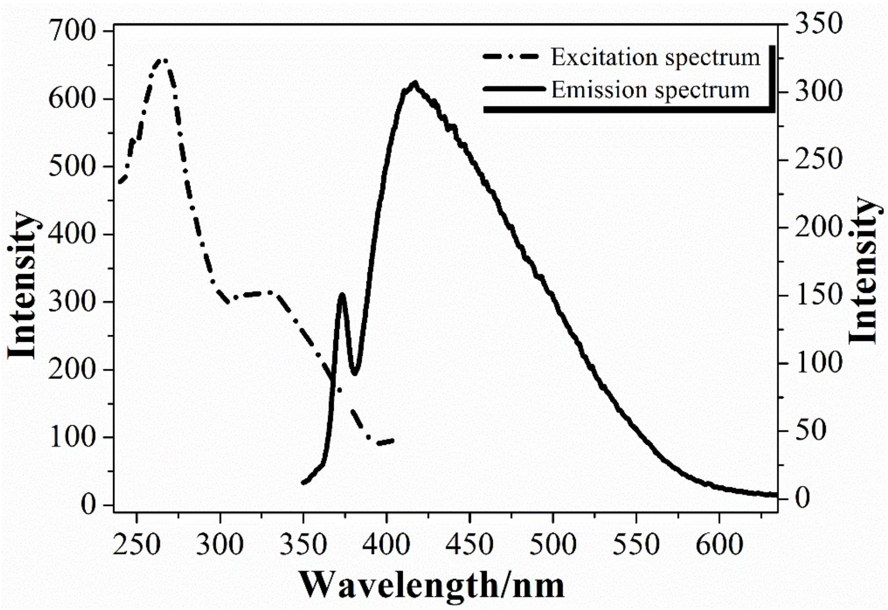

Fig. S2 Fluorescence excitation and emission spectra of TPEDB in alkaline medium.

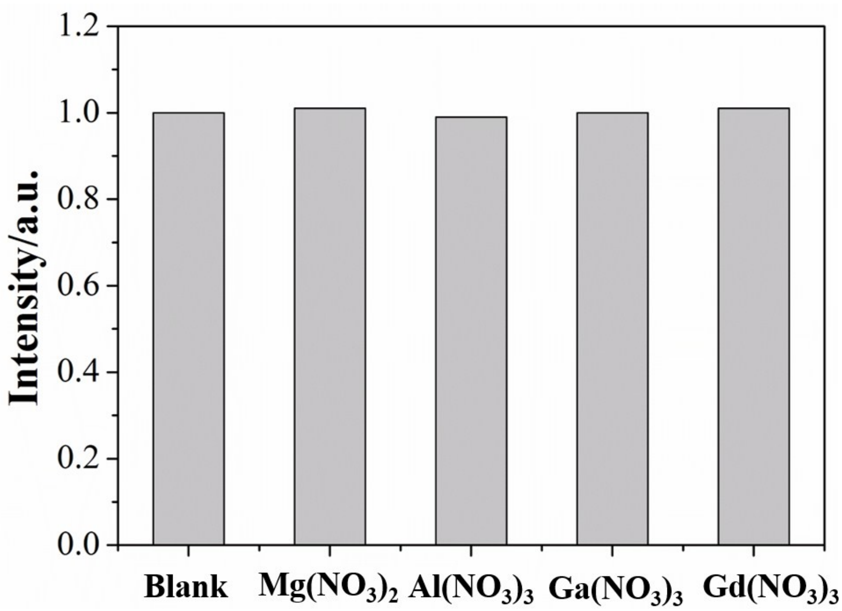

Fig. S3 Fluorescence emission intensities of TPEDB with the absence/presence of precursors for LDHs.

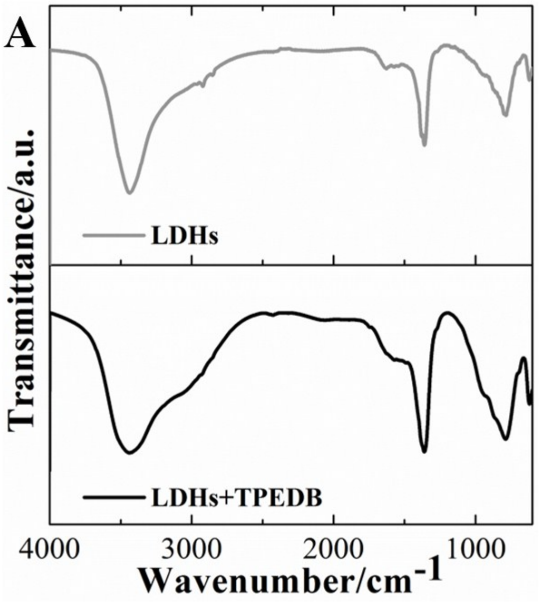

Fig. S4 Normalized IR transmittance of (A) LDHs and (B) MMT in the presence/absence of TPEDB in the range of 600–4000 cm^{-1}.

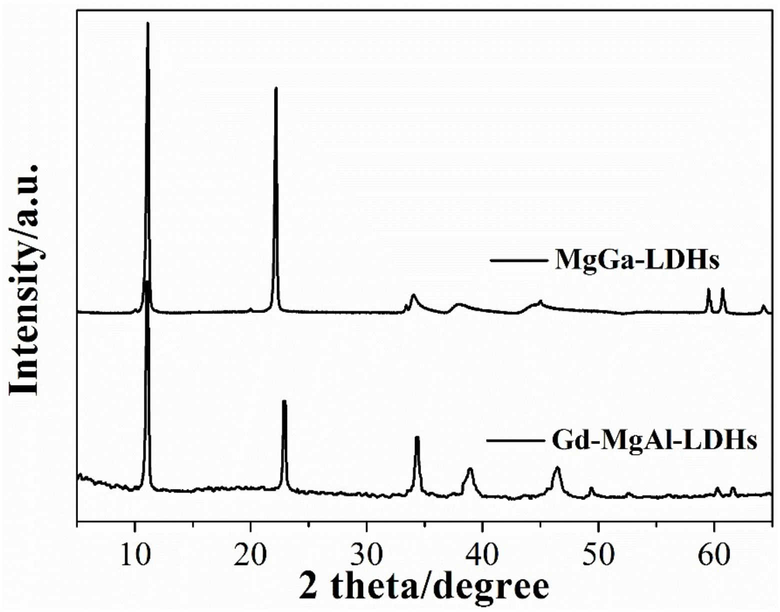

Fig. S5 XRD patterns of MgGa-LDH and Gd-doped MgAl-LDHs.

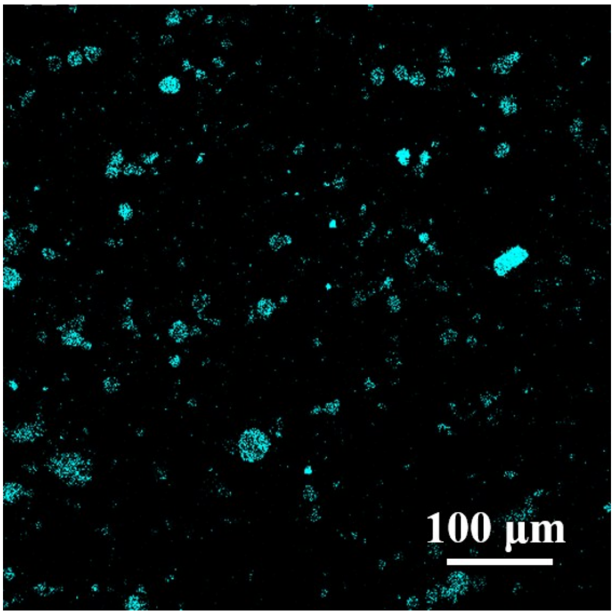

Fig. S6 Fluorescence confocal microscopy image of PE-5% LDH film treated with TPEDB.

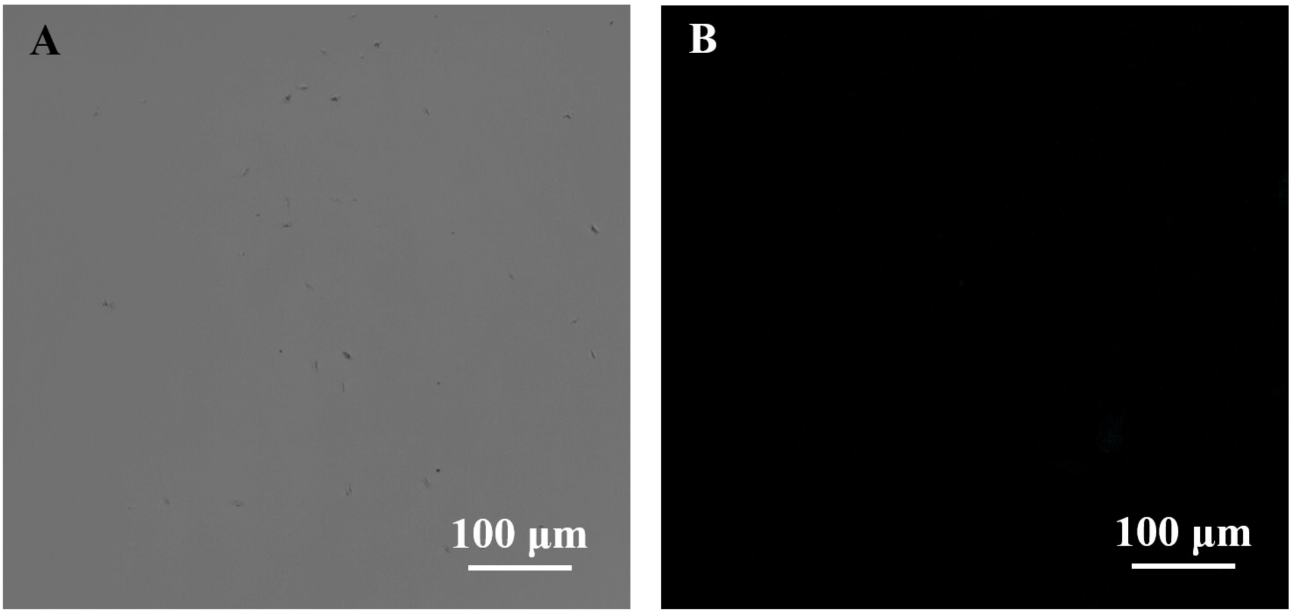

Fig. S7 Fluorescence confocal microscopy images of pure PE matrix treated with TPEDB for (A) bright-field and (B) fluorescent images.

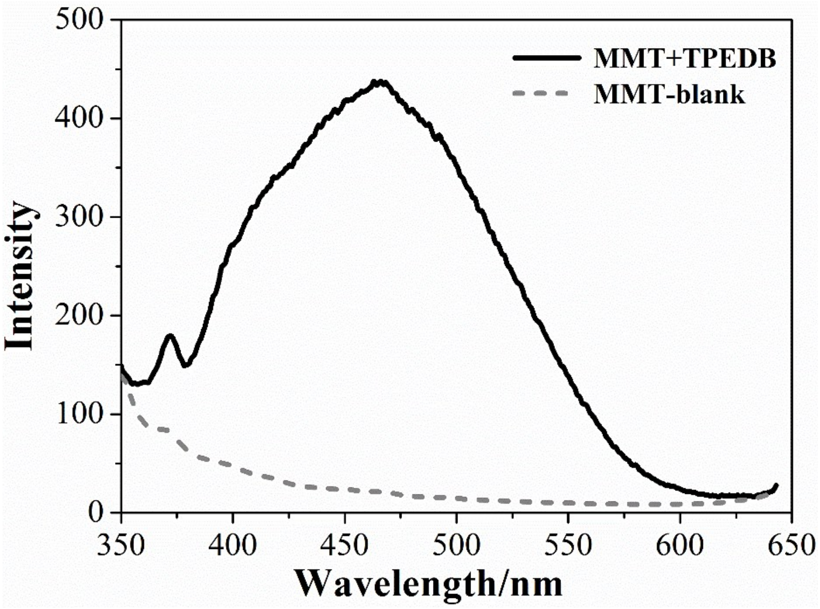

Fig. S8 Fluorescence emission spectra of MMT in the presence/absence of TPEDB.

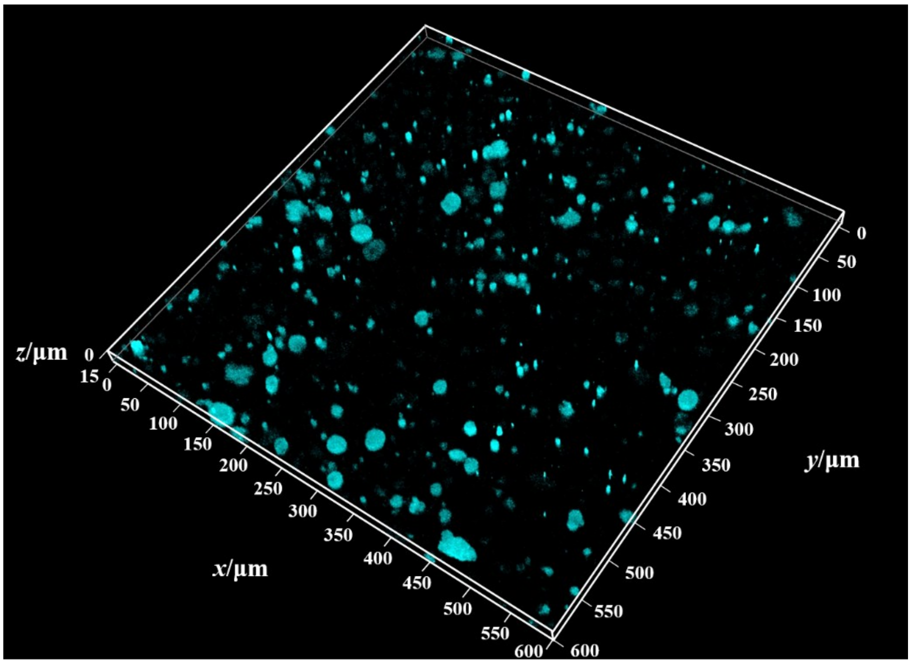

Fig. S9 3D representation of fluorescence microscopy image for 5% MMT (cyan colored patterns) in PE matrix after the staining of TPEDB.

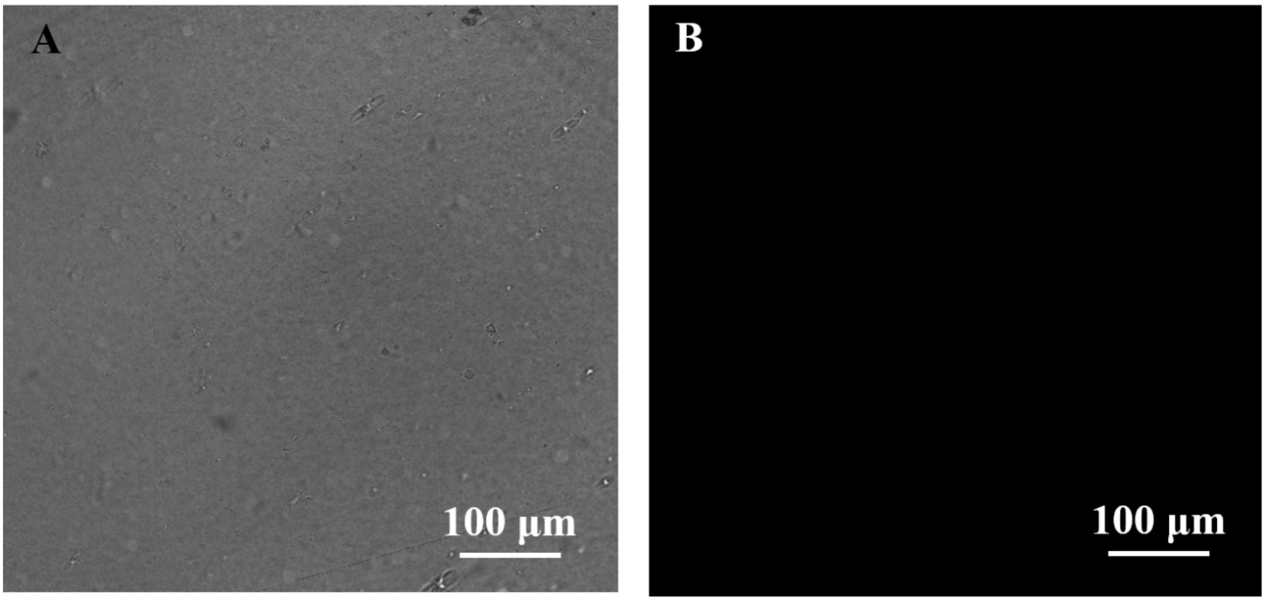

Fig. S10 Fluorescence confocal microscopy images of pure PP matrix treated with TPEDB for (A) bright-field and (B) fluorescent images.

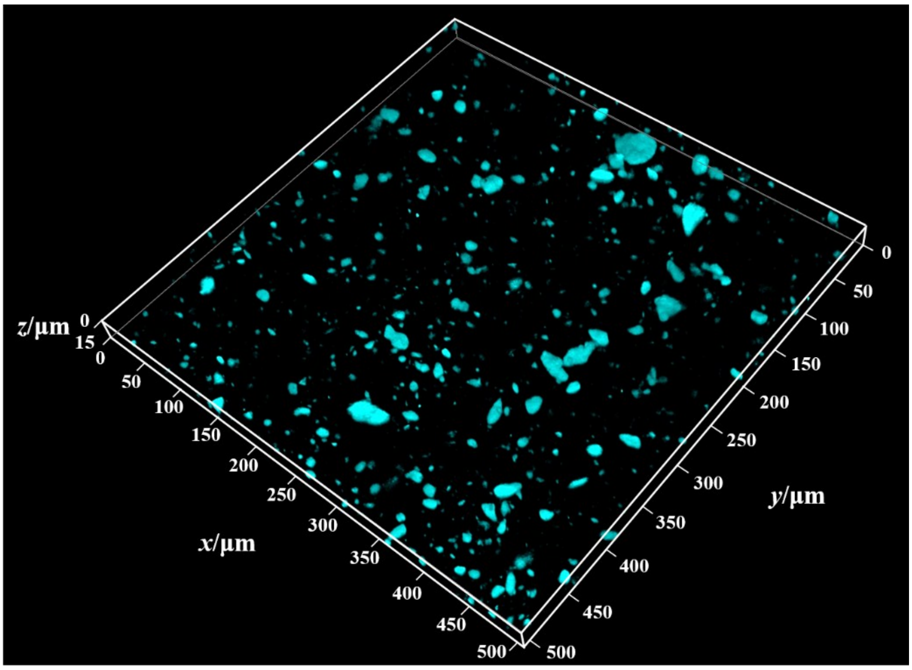

Fig. S11 3D representation of fluorescence microscopy image for 5% LDHs (cyan colored patterns) in PP matrix after the staining of TPEDB.

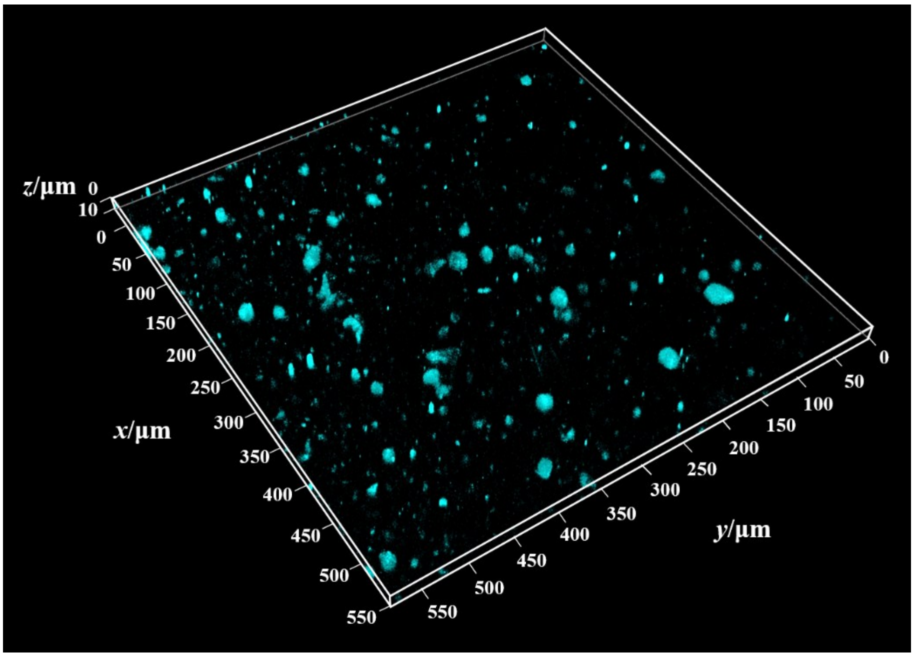

Fig. S12 3D representation of fluorescence microscopy image for 5% MMT (cyan colored patterns) in PP matrix after the staining of TPEDB.

References

(1) L. Bai, N. Xue, X. Wang, W. Shi and C. Lu, *Nanoscale*, 2017, **9**, 6658-6664.

(2) S. Li, J. Lu, M. Wei, D. G. Evans and X. Duan, *Adv. Funct. Mater.*, 2010, **20**, 2848-2856.

(3) D. Yan, J. Lu, J. Ma, S. Qin, M. Wei, D. G. Evans and X. Duan, *Angew. Chem. Int. Ed.*, 2011, **50**, 7037-7040.

(4) Y. Liu, C. Deng, L. Tang, A. Qin, R. Hu, J. Z. Sun and B. Z. Tang, *J. Am. Chem. Soc.*, 2011**,** **133**, 660-663.

(5) W. Zhou, Y. Cao, D. Sui and C. Lu, *Angew. Chem. Int. Ed.*, 2016, **55**, 4236-4241.

(6) M. H. N. Yio, M. J. Mac, H. S. Wong and N. R. Buenfeld, *J. Microsc.*, 2015, **258**, 151−169.
